# Effects of Ramadan fasting on platelet reactivity in diabetic patients treated with clopidogrel

**DOI:** 10.1186/s12959-017-0138-0

**Published:** 2017-06-02

**Authors:** W. Bouida, H. Baccouche, M. Sassi, Z. Dridi, T. Chakroun, I. Hellara, R. Boukef, M. Hassine, F. Added, R. Razgallah, I. Khochtali, S. Nouira, On behalf of the Ramadan Research Group

**Affiliations:** 1 Emergency Department, Fattouma Bourguiba University Hospital, 5000 Monastir, Tunisia; 2 Laboratory of Biology, Maternity and Neonatal Medicine Center, 5000 Monastir, Tunisia; 3 Cardiology Department, Fattouma Bourguiba University Hospital, 5000 Monastir, Tunisia; 4 Regional Blood Transfusion Center, Farhat Hached University Hospital, 4004 Sousse, Tunisia; 5 Hematology Department, Fattouma Bourguiba University Hospital, 5000 Monasitr, Tunisia; 6 Emergency Department, Sahloul University Hospital, 4011 Sousse, Tunisia; 7 Cardiology Department, Abderrahman Mami University Hospital, 1080 Ariana, Tunisia; 8 Endocrinology and Internal Medicine Department, Fattouma Bourguiba University Hospital, 5000 Monastir, Tunisia; 9 Medis Laboratories, 1053 Tunis, Tunisia; 10 Research Laboratory (LR12SP18), University of Monastir, 5000 Monastir, Tunisia

**Keywords:** Fasting, Platelet aggregation inhibitors, Diabetes mellitus, Platelet activation, Clopidogrel

## Abstract

**Background:**

The effects of Ramadan fasting (RF) on clopidogrel antiplatelet inhibition were not previously investigated. The present study evaluated the influence of RF on platelet reactivity in patients with high cardiovascular risk (CVR) in particular those with type 2 diabetes mellitus (DM).

**Methods:**

A total of 98 stable patients with ≥2 CVR factors were recruited. All patients observed RF and were taking clopidogrel at a maintenance dose of 75 mg. Clinical findings and serum lipids data were recorded before Ramadan (Pre-R), at the last week of Ramadan (R) and 4 weeks after the end of Ramadan (Post-R). During each patient visit, nutrients intakes were calculated and platelet reactivity assessment using Verify Now P2Y12 assay was performed.

**Results:**

In DM patients, the absolute PRU changes from baseline were +27 (*p* = 0.01) and +16 (*p* = 0.02) respectively at R and Post-R. In addition, there was a significant increase of glycemia and triglycerides levels with a significant decrease of high-density lipoprotein. In non DM patients there was no significant change in absolute PRU values and metabolic parameters. Clopidogrel resistance rate using 2 cut-off PRU values (235 and 208) did not change significantly in DM and non DM patients.

**Conclusions:**

RF significantly decreased platelet sensitivity to clopidogrel in DM patients during and after Ramadan. This effect is possibly related to an increase of glycemia and serum lipids levels induced by fasting.

**Trial registration:**

Clinical Trials.gov NCT02720133. Registered 24 July 2014.Retrospectively registered.

## Background

Each year, during the Ramadan month, millions of Muslims with cardiovascular risk factors observe obligatory fasting from early down to dusk. Ramadan fasting (RF) has been shown to be associated with vascular and metabolic disorders including glycemic control and lipid profile [1–3]. It may also alter pharmacologic properties of some medications resulting from the change in eating patterns and physiologic parameters disturbances [4–8]. Although patients with coronary artery disease (CAD) under antiplatelet therapy may be exempted from RF, many of them still insist to observe strictly their fasting. Clopidogrel is widely used and plays a pivotal role in reducing recurrent thrombotic events in patients with CAD, but the wide response variability of patients to this agent could lead to pharmacodynamics failure [9, 10]. Clopidogrel resistance has been documented in the range of 5–44% across the world and has been associated with adverse thrombotic events [11–13]. This is particularly true in patients type 2 with diabetes mellitus (DM) known for their suboptimal response to antiplatelet agents [14–16]. Consequently, accurate assessment of clopidogrel response during RF may have potential implications with regard to the delicate balance between thrombosis and bleeding in the monitoring of patients under antiaggregating agents. Investigating this issue is now possible as several assays are available to measure platelet reactivity in order to better predict ischemic and/or bleeding complications [17]. Currently, it is not clear whether RF affects platelet reactivity in patients already treated with clopidogrel. This study was planned to assess the effects of RF on clopidogrel resistance in patients at high cardiovascular risk and especially those with DM.

## Methods

### Participants

This was a prospective observational study that was carried out in a group of patients having at least two currently accepted cardiovascular risk factors classification [18]. Patients were recruited from academic and non-academic medical centers serving a population of 500.000 Tunisian inhabitants. Participants were screened in outpatient clinics (cardiology, endocrinology, internal medicine, family medicine) when they presented for scheduled follow-up. Selection was based on the participant’s decision to fast, while taking clopidogrel therapy for at least 6 months. Exclusion criteria included patients under 40 years or those with unstable diabetes, acute coronary syndrome within the past year prior to enrollment, current or previous (14 days) use of glycoprotein IIb/IIIa, inability to give informed consent, baseline platelet count <100 × 10^6^/L, current use of antidepressants, and chronic disease with <1 year expected mortality. The study was approved by the Institutional Review Board of Fattouma Bourguiba University Hospital and all patients provided written informed consent. After screening, the study design and requirements were thoroughly explained to the participants.

### Methods

The study was conducted during 4 years (2010–2014) with three separate assessment visits in each year: 1) the last week before Ramadan (Pre-R) which represented the baseline period; 2) the last week of Ramadan (R); 3) and during the last week of the month following Ramadan (Post-R). Each patient served as his own control and was required to take the prescribed clopidogrel dose daily and chart the intake in a dosing diary. The duration of fasting was approximately 12 h from sunrise to sunset (the time of abstinence from food) during a 30 day period. The assessment in each of the three visits involved clinical exam and blood sampling for hematologic and metabolic tests.

#### Clinical assessment

Body weight and height were performed by a well-trained staff member. Weight was measured while the subjects were minimally clothed without shoes using digital scales and recorded to the nearest 0.1 kg. Body mass index (BMI) was calculated as body weight (kg) divided by squared height in meters (m2). Physical examination was carried out in all participants including systolic (SBP), diastolic (DBP) blood pressure, and heart rate. The visit is completed by a questionnaire on diet beginning 2 days before the blood sampling. No special nutritional regimen was applied to the participants during the study. All subjects were encouraged to continue their usual lifestyle and activities. The rate of hypoglycemic (symptomatic and non-symptomatic) and hyperglycemic episodes requiring ED admission was recorded within the three periods of the study. Hypoglycemia was defined as blood glucose <3.5 mmol/l. Compliance to current treatment (clopidogrel, oral hypoglycemic agents, statins…) was assessed by the attending physician based on interview and pill count. Venous blood samples were collected from the enrolled participants during the three time points. The time of blood sampling in the study was 9–10 a.m., at which all participants were fast. For the purpose of the study, we asked our patients to take clopidogrel treatment as late as possible. As Ramadan month during the study period has coincided with summer season, clopidogrel was generally taken between midnight and 1 am. We added this detail in the paper.

#### Hematological parameters and clopidogrel response assays

Blood samples were analyzed directly for hemoglobin, hematocrit, and platelet cell count. Prothrombin time and (PT) activated partial thromboplastin time (APTT) were studied in fresh samples. Platelet reactivity was assessed by the Verify Now P2Y12 point-of-care assay (Accumetrics, San Diego, CA, USA) using venous blood samples collected in tubes containing 3.2% sodium citrate. Verify Now P2Y12 specifically evaluates clopidogrel effect on P2Y12 receptor by optical turbidimetry. Results are reported as P2Y12 reaction units (PRU); the lower the PRU value the higher the platelet aggregation inhibition by clopidogrel. High platelet reactivity after clopidogrel (clopidogrel resistance) was defined at two cutoff values (PRU ≥ 235 [19] and ≥208 [20]). Reading recorded by the study team was not revealed to patients and their primary physician.

#### Metabolic measurements

An automated analyzer (Beckman Coulter DXC 600, UK) measured the concentrations of biochemical parameters using the appropriate reagents (Beckman Coulter, UK). Glucose, uric acid, total cholesterol (TC) and triglycerides (TG) were determined using an enzymatic colorimetric method (glucose oxidase, uricase, lipoprotein lipase-glycerol kinase reactions, cholesterol esterase-cholesteroloxidase reactions, respectively).High-density lipoprotein cholesterol (HDL-C) concentrations were determined by immuno-inhibition. Low-density lipoprotein cholesterol (LDL-C) was calculated using the Friedewald formula: LDL-C (mmol/L) = TC – HDL-C – TG: 2.2.

### Statistical analysis

All continuous data are presented as either the median with interquartile range (IQR) or the mean with SD according to the distribution of the data. The categorical data are presented as the percentage frequency of occurrence. The Kolmogorov-Smirnov test was performed to assess the normal distribution. Each subject served his own control by comparing his/her values before Ramadan with those during and after Ramadan. Differences between results were analyzed using paired samples t test for normally distributed parameters and Wilcoxon signed Rank test for not normally distributed parameters. Statistical significance was considered at *p* < 0.05 for all tests. Comparison was performed between patients with and without DM. Statistical analyses were conducted by using SPSS statistical software (version 11.5, SPSS Inc. Chicago, IL).

## Results

**Fig. 1:**
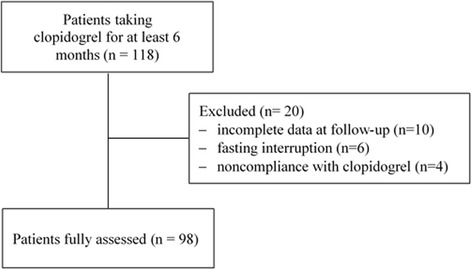
Study profile

**Fig. 2:**
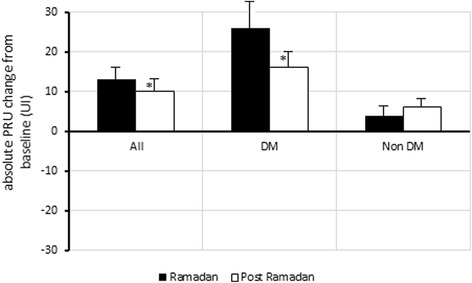
Median of absolute PRU change from baseline during and after Ramadan. *p < 0.05 between Ramadan and Post-Ramadan

One hundred eighteen patients under clopidogrel were included. From these, 20 patients (16.9%) were excluded from the analysis due to incomplete data at follow-up (*n* = 10), stop fasting (*n* = 6), and noncompliance with clopidogrel treatment (*n* = 4). At the completion of the study, 98 participants had been followed up throughout the study (Fig. 1). Demographic and clinical characteristics of the participants are summarized in Table 1. The mean age was 59.1 ± 10 years and 87,7% were men (*n* = 86). Most of the participants had at least 2 to 3 cardiovascular risk factors mainly dyslipidemia (77.5%) and DM (64.3%). Dual therapy with clopidogrel and aspirin was prescribed in 90 patients (91.8%) (Table 1). Mean blood pressure and heart rate did not change significantly between the three periods. Weight and BMI decreased significantly during RF and returned to baseline values at post-R period (Table 2). Caloric intake decreased slightly during RF and increased thereafter but all00these changes were not significant as was the distribution of caloric intake between glucids, lipids and proteins. Mean time intervals between clopidogrel taking and Verify Now testing was 9 ± 1 h at pre-R, 10 ± 1 h at R, and 10.5 ± 2 h at post-R. The time intervals were similar for the three visits (*p* = 0.68). Results of platelet reactivity at each time point for each period are presented in Table 3. Overall, PRU values increased significantly from pre-R to R and post-R periods (absolute increase +13 and +11 respectively; *p* = 0.03). In patients with DM, the absolute increase of PRU values from baseline was +27 during RF (*p* = 0.01) and +16 at post-R period (*p* = 0.02) (Fig. 2). Conversely, in non DM participants changes of PRU values were not significant between the three periods. In the overall group, the rate of clopidogrel resistance did not change significantly between the three periods whether using a PRU cutoff at 235 or 208 (Table 3). Using the cut off 208, the rate of patients with DM who were resistant during Ramadan and post-Ramadan periods compared with baseline was respectively 60.3% and 55.5 vs 52.3%, (*p* = 0.22). In the overall population no significant differences were observed during the three periods regarding hemoglobin, hematocrit, platelet count, prothrombin time, and activated partial thromboplastin time (Table 4). Glycemia was significantly higher during Ramadan (10.4 ± 4.7 mmol/L) compared to baseline (9.6 ± 4.9 mmol/L) (*p* = 0.003). Glycemia decreased after Ramadan fasting to 9.9 ± 4.8 mmol/L. Similar changes of glycemia were observed in patients with DM. With regard to serum lipid in DM patients, the following changes were observed: serum TG levels also increased significantly from 1.65 ± 0.87 mmol/L at baseline to 2.26 ± 1.91 mmol/L at Ramadan period (*p* = 0.002) and 1.74 ± 0.90 at post-R period (*p* = 0.01); HDL cholesterol decreased during Ramadan period from 1.01 ± 0.27 mmol/l at baseline to 0.93 ± 0.22 mmol/L (*p* = 0.001) during Ramadan, and returned to baseline values at post Ramadan period (1.02 ± 0.15 mmol/l). Serum lipids did not change significantly in non-DM patients. The other metabolic parameters (serum cholesterol, LDL cholesterol and uric acid) did not show significant changes between the three periods in patients with and without DM (Table 4). Non-symptomatic hypoglycemic events were reported in one participant before Ramadan, in three participants during Ramadan (two in DM and one in non DM patients), and in two participants after Ramadan. None of these events required ED admission. No participant was hospitalized for hyperglycemic complication.

**Table 1:** Baseline Characteristics

	Total *n* = 98
Age years (mean ± SD)	59.1 ± 10
Male gender; *n* (%)	86 (87.7%)
Cardiovascular risk factors *n* (%)	
Dyslipidemia	76 (77.5)
Diabetes	63 (64.3)
Arterial hypertension	61 (62.2)
Smoking	58 (59.2)
Coronary artery disease	48 (48.9)
Number	
2	60 (61.2)
3	27 (27.5)
≥ 4	11 (11.3)
Treatment *n* (%)	
Aspirin	90 (91.8)
Statins	81 (82.6)
Oral antidiabetics	63 (64.2)
Enzyme converting inhibitors	59 (60.2)
Beta-blockers	42 (42.8)
Diuretics	20 (20.4)
Angiotension receptor antagonists	12 (12.2)
Vitamin K antagonists	5 (4.9)
Clopidogrel indications	
Coronary artery disease	90 (91.8)
Peripheral artery disease	8 (8.2)

**Table 2:** Clinical and caloric intake changes during the three protocol periods

	Pre-Ramadan mean (SD)	Ramadan mean (SD)	Post-Ramadan mean (SD)
Systolic arterial pressure (mmHg)	139 (24)	137 (24)	136 (24)
Diastolic arterial pressure (mmHg)	79 (12)	77 (12)	78 (12)
Pulse (b/min)	78 (12)	81 (14)	79 (14)
Weight (kg)	83.2 (11.2)	81.7 (11.1)^*^	82.9 (13.9)^£^
Body mass index (kg/m^2^)	29.5 (3.7)	29.0 (3.6)^*^	29.6 (3.7)^£^
Caloric total intake (kcal/j)	2156 (449)	2035 (455)	2209 (551)
Carbohydrate intake (%)	55.2 (8.3)	56.8 (7.3)	55.1 (8.8)
Protein intake (%)	17.4 (3.9)	17.3 (3.4)	17.3 (4.8)
Fat intake (%)	27.4 (7.8)	25.7 (6.7)	27.6 (8.3)

^*^ *p* < 0.05 between Pre-Ramadan and Ramadan, ^£^ *p* < 0.05 between Ramadan and post-Ramadan

**Table 3:** Platelet reactivity and clopidogrel resistance in patients with and without diabetes mellitus

	All	DM	Non DM
	*n* = 98	*n* = 63	*n* = 35
Pre-Ramadan			
PRU median (IQR)	199 (157–251)	200 (157–253)	196 (157–248)
Clopidogrel resistance *n* (%)			
PRU > 235	36 (36.7)	23 (36.5)	13 (37.1)
PRU > 208	48 (48.9)	33 (52.3)	15 (42.8)
Ramadan			
PRU median (IQR)	212 (169–257)	227 (176–261)^*^	200 (159–252) ^£^
Clopidogrel resistance *n* (%)			
PRU > 235	39 (39.7)	27 (42.8)	12 (34.2)
PRU > 208	54 (55.1)	38 (60.3)	16 (45.7)
Post-R (*n* = 109)			
PRU median (IQR)	210 (166–251)	216 (176–247)^*^	202 (153–254)
Clopidogrel resistance *n* (%)			
PRU > 235	39 (39.7)	23 (36.5)	16 (45.7)
PRU > 208	52 (53.0)	35 (55.5)	17 (48.5)

*DM* diabetes mellitus

^*^ *p* < 0.05 compared to Pre-Ramadan.

^£^ *p* < 0.05 compared to patients with DM

**Table 4:** Biological changes during the three protocol periods

	All			Patients with DM *n* = 63			Patients without DM *n* = 35		
	Pre-Ramadan mean (SD)	Ramadan mean (SD)	Post-Ramadan mean (SD)	Pre-Ramadan mean (SD)	Ramadan mean (SD)	Post-Ramadan mean (SD)	Pre-Ramadan mean (SD)	Ramadan mean (SD)	Post-Ramadan mean (SD)
Hematological									
Hemoglobin g/dl	13.3 (1.2)	13.6 (1.5)	13.4 (1.3)	13.1 (1.1)	13.3 (1.3)	13.1 (1.2)	13.8 (1.1)	14.1 (1.4)	14.0 (1.1)
Hematocrit %	41 (5)	41 (4)	40 (4)	41 (4)	40 (4)	39 (4)	42 (7)	42 (4)	41 (4)
Platelets count × 10^3^/ml	216 (22)	214 (21)	216 (24)	214 (24)	212 (24)	209 (23)	214 (22)	216 (23)	225 (22)
Prothrombin time (sec)	10.8 (0.6)	10.9 (0.6)	10.9 (0.5)	10.9 (0.5)	11.1 (0.5)	11.1 (0.6)	11.1 (0.7)	11.0 (0.5)	10.9 (0.5)
APTT (sec)	36.6 (1.6)	37.0 (1.8)	37.4 (1.7)	38.0 (2.5)	39.9 (4.2)	40.7 (4.7)	39.2 (2.5)	40.1 (3.8)	39.0 (2.8)
Biochemical									
Glycemia mmol/l	9.6 (4.9)	10.4 (4.7)^*^	9.9 (4.8)	11.40 (5.05)	12.23 (4.81)^*^	11.50 (4.77)	6.49 (2.39)	7.04 (1.55)	6.78 (3.01)
Cholesterol mmol/l	3.82 (1.19)	3.87 (1.26)	3.90 (1.21)	3.76 (1.13)	3.82 (1.33)	3.86 (1.21)	4.02 (1.35)	4.15 (1.19)	4.28 (1.24)
Triglycerides mmol/l	1.76 (1.04)	2.23 (1.16)^*^	1.90 (1.17)^£^	1.65 (0.87)	2.26 (1.91)^*^	1.74 (0.90)^£^	1.95 (1.28)	2.16 (1.46)	2.20 (1.52)
LDL cholesterol mmol/l	2.02 (0.97)	2.98 (0.97)	2.0 (0.98)	1.86 (0.87)	1.99 (0.87)	2.06 (0.95)	2.12 (1.20)	2.20 (1.06)	2.25 (1.06)
HDL cholesterol mmol/l	1.01 (0.27)	0.93 (0.22)^*^	1.02 (0.15)^£^	1.01 (0.29)	0.90 (0.23)^*^	0.97 (0.27)	1.01 (0.23)	1.0 (0.2)	1.05 (0.23)

*DM* diabetes mellitus, ^*^ *p* < 0.05 between Pre-Ramadan and Ramadan, ^£^ *p* = 0.001between Ramadan and Post-Ramadan

*APTT* activated partial thromboplastin time, *LDL/HDL* low-density/high-density lipoprotein

## Discussion

Our results showed that platelet reactivity increased significantly during RF essentially in patients with DM and persisted 1 month later. These effects were associated with a significant increase in glycemia and serum TG levels and decrease of HDL cholesterol. In patients without DM, no significant changes were observed. No significant clinical event related to RF was reported during this study.

Antiplatelet agents are one of the most frequently used drugs in clinical practice. With regard to their wide pharmacodynamic variability, RF could significantly modify the response to these drugs. Multiple factors including changes in glycemic control and lipid profile may influence platelet reactivity and response to antiaggregating agents during RF. Patients with DM are particularly exposed to this hazard given their adverse metablolic features and comorbidities that could affect platelet function [21, 22]. Although the mechanisms for clopidogrel resistance related to RF are probably multiple in diabetic patients, inadequate metabolic control might be one of the contributor factors [23, 24]. Major glycemic excursions associated with RF may lead to non-enzymatic glycosylation of platelet membrane proteins changing their structure and conformation and consequently their function [25, 26]. Hyperglycemia may also affect platelet clopidogrel response through an increase of superoxide production or inflammatory markers discharge [20]. Geisler et al. [27] reported that diabetic patients with hyperglycemia had increased amounts of inflammatory markers in comparison to normoglycemics and non-diabetic patients. They showed that higher levels of inflammatory markers correlated with decreased response to aspirin and clopidogrel dual therapy, and found that hyperglycemia positively correlated with increased thrombus formation. In the present study, we showed that higher PRU values related to fasting was associated with a significant increase of serum triglycerides and decrease of HDL cholesterol which suggest that RF may have a lipid-related prothrombotic action. The fact that these parameters increased in the same time does not prove of course that the higher PRU values are caused by metabolic changes during Ramadan. Decrease in fish and olive oil consumption with increase of fatty acids mobilization from adipose tissue during RF could have a detrimental effects on serum lipid composition and may contribute to promote suboptimal response to antiplatelet agents. Although we demonstrated an increase of PRU values during and after RF, we did not observe higher rate of clopidogrel resistance as defined by the two cut-offs currently accepted. Early studies suggested that optimal threshold is between 230 and 240 PRU [28, 29], while post-hoc analysis of GRAVITAS suggested a somewhat lower cut-off, 208 PRU [30]. In our study, we used both PRU values and we demonstrated similar results and a trend to award a resistance increase with RF in DM patients. As optimal antiplatelet inhibition is essential in DM patients with CAD, we believe that those with borderline PRU values should be considered at increased risk of clopidogrel resistance during and after RF and should be managed on this basis.

## Limitations

First, the number of DM patients is almost twice the number of non-DM patients. The fact that no differences in platelet reactivity during Ramadan fasting found in non-DM patients, could be explained by the lower number of patients. Of note, predominance of patients with DM could be expected since many participants were recruited from outpatient endocrinology clinic.

Second, although we attempted to verify compliance to clopidogrel and the treatment regimens during the three study periods, we cannot absolutely rule out inadequate compliance. Third, only the VerifyNow P2Y12 assay was used in our study to evaluate platelet function. We should note that except for a few, there are no head-to-head comparison studies between the most commonly used tests. Based on available evidence, diagnostic performance of VerifyNow assays is comparable to light transmission aggregometry which is the most widely accepted test of platelet function both in terms of biological and clinical endpoints. In addition, the Verify Now was validated in sufficiently large sample size for prediction of stent thrombosis and bleeding which justify our choice. Finally, this pilot study was not designed (size, limited follow up) to assess associations with clinical outcomes. Larger prospective studies may be warranted to elucidate the clinical regenace of our findings.

Hence, the clinical relevance of our results is unknown. Specific clinical studies are needed to define whether the decrease of clopidogrel antiplatelet activity may provide biological support for RF detrimental outcome of RF in diabetic patients.

## Conclusions

In conclusion, the present study demonstrated that RF could induce an increase in clopidogrel resistance that seems to be related to a transient disturbance of glycemic control and lipid profile. The selective decrease response to antiplatelet agents during RF in diabetic patients in our study means that this population are at increased risk. This population might benefit from diagnostic testing of platelet function for whom we should better control lipid and glucose levels to adapt the dose of current medications such as statins and antidiabetics.

## Abbreviations

APTT: Activated partial thromboplastin time
BMI: Body mass index
CAD: Coronary artery disease
CVR: Cardiovascular risk
DBP: Diastolic blood pressure
DM: Diabetes mellitus
HDL-C: High-density lipoprotein cholesterol
LDL-C: Low-density lipoprotein cholesterol
Post-R: After Ramadan
Pre-R: Before Ramadan
PRU: P2Y12 reaction units
PT: Prothrombin time
R: Ramadan
RF: Ramadan fasting
SBP: Systolic blood Pressure
TC: Total cholesterol
TG: Triglycerides

## References

[1] Barkia A, Mohamed K, Smaoui M, Zouari N, Hammami M, Nasri M (2011). Change of diet, plasma lipids, lipoproteins, and fatty acids during Ramadan: a controversial association of the considered Ramadan model with atherosclerosis risk. J Health Popul Nutr.

[2] Benaji B, Mounib N, Roky R, Aadil N, Houti IE, Moussamih S, et al. Diabetes and Ramadan: review of the literature. Diabetes Res Clin Pract. 2006;73:117–25. 10.1016/j.diabres.2005.10.028 16647781

[3] Salti I, Bénard E, Detournay B, Bianchi-Biscay M, Le Brigand C, Voinet C, et al., EPIDIAR study group. A population-based study of diabetes and its characteristics during the fasting month of Ramadan in 13 countries: results of the epidemiology of diabetes and Ramadan 1422/2001 (EPIDIAR) study. Diabetes Care. 2004;27:2306–11. 10.2337/diacare.27.10.2306 15451892

[4] Aslam M, Assad A (1986). Drug regimens and fasting during Ramadan: a survey in Kuwait. Public Health.

[5] Rashed AH (1992). The fast of Ramadan. BMJ.

[6] Addad F, Amami M, Ibn Elhadj Z, Chakroun T, Marrakchi S, Kachboura S (2014). Does Ramadan fasting affect the intensity of acenocoumarol-induced anticoagulant effect?. Br J Haematol.

[7] Lai YF, Cheen MH, Lim SH, Yeo FH, Nah SC, Kong MC, et al. The effects of fasting in Muslim patients taking warfarin. J Thromb Haemost. 2014;12:349–54. 10.1111/jth.12496 24354801

[8] Farooq S, Nazar Z, Akhtar J, Irfan M, Subhan F, Ahmed Z, et al. Effect of fasting during Ramadan on serum lithium level and mental state in bipolar affective disorder. Int Clin Psychopharmacol. 2010;25:323–7. 10.1097/YIC.0b013e32833d18b2 20827213

[9] Sharma RK, Voelker DJ, Sharma R, Reddy HK, Dod H, Marsh JD (2012). Evolving role of platelet function testing in coronary artery interventions. Vasc Health Risk Manag.

[10] Tantry US, Gesheff M, Liu F, Bliden KP, Gurbel PA (2014). Resistance to antiplatelet drugs: what progress has been made?. Expert Opin Pharmacother.

[11] Siller-Matula JM, Trenk D, Schrör K, Gawaz M, Kristensen SD, Storey RF, et al., EPA (European Platelet Academy). Response variability to P2Y12 receptor inhibitors: expectations and reality. JACC Cardiovasc Interv. 2013;6:1111–28. 10.1016/j.jcin.2013.06.011 24262612

[12] Gurbel PA, Bliden KP, Hiatt BL, O'Connor CM (2003). Clopidogrel for coronary stenting: response variability, drug resistance, and the effect of pretreatment platelet reactivity. Circulation.

[13] Matetzky S, Shenkman B, Guetta V, Shechter M, Beinart R, Goldenberg I, et al. Clopidogrel resistance is associated with increased risk of recurrent atherothrombotic events in patients with acute myocardial infarction. Circulation. 2004;109:3171–5. 10.1161/01.CIR.0000130846.46168.03 15184279

[14] Schuette C, Steffens D, Witkowski M, Stellbaum C, Bobbert P, Schultheiss HP, et al. The effect of clopidogrel on platelet activity in patients with and without type-2 diabetes mellitus: a comparative study. Cardiovasc Diabetol. 2015;14:15. 10.1186/s12933-015-0182-7 PMC4324649 25645908

[15] Angiolillo DJ, Suryadevara S (2009). Aspirin and clopidogrel: efficacy and resistance in diabetes mellitus. Best Pract Res Clin Endocrinol Metab.

[16] Price MJ (2014). Diabetes mellitus and clopidogrel response variability. J Am Coll Cardiol.

[17] Aradi D, Collet JP, Mair J, Plebani M, Merkely B, Jaffe AS, et al., Study Group on Biomarkers in Cardiology of the Acute Cardiovascular Care Association of the European Society of Cardiology, Working Group on Thrombosis of the European Society of Cardiology. Platelet function testing in acute cardiac care - is there a role for prediction or prevention of stent thrombosis and bleeding? Thromb Haemost. 2015;113:221–30. 10.1160/TH14-05-0449 25413597

[18] Grundy SM, Pasternak R, Greenland P, Smith S, Fuster V (1999). Assessment of cardiovascular risk by use of multiple-risk-factor assessment equations: a statement for healthcare professionals from the American Heart Association and the American College of Cardiology. Circulation.

[19] Price MJ, Endemann S, Gollapudi RR (2008). Prognostic significance of post-clopidogrel platelet reactivity assessed by a point-of-care assay on thrombotic events after drug-eluting stent implantation. Eur Heart J.

[20] Price M, Angiolillo D, Teirstein P (2011). Platelet reactivity and cardiovascular outcomes after percutaneous coronary intervention: a time dependent analysis of the Gauging Responsiveness with a VerifyNow P2Y12 assay: impact on thrombosis and safety (GRAVITAS) trial. Circulation.

[21] Ferroni P, Basili S, Falco A, Davì G (2004). Platelet activation in type 2 diabetes mellitus. J Thromb Haemost.

[22] Grant PJ (2007). Diabetes mellitus as a prothrombotic condition. J Intern Med.

[23] Demirtunc R, Duman D, Basar M, Bilgi M, Teomete M, Garip T (2009). The relationship between glycemic control and platelet activity in type 2 diabetes mellitus. J Diabetes Complicat.

[24] Singla A, Antonino MJ, Bliden KP, Tantry US, Gurbel PA (2009). The relation between platelet reactivity and glycemic control in diabetic patients with cardiovascular disease on maintenance aspirin and clopidogrel therapy. Am Heart J.

[25] Watala C, Golanski J, Pluta J, Boncler M, Rozalski M, Luzak B, et al. Reduced sensitivity of platelets from type 2 diabetic patients to acetylsalicylic acid (aspirin)-its relation to metabolic control. Thromb Res. 2004;113:101–13. 10.1016/j.thromres.2003.12.016 15115665

[26] Winocour PD, Watala C, Perry DW, Kinlough-Rathbone RL (1992). Decreased platelet membrane fluidity due to glycation or acetylation of membrane proteins. Thromb Haemost.

[27] Geisler T, Mueller K, Aichele S, Bigalke B, Stellos K, Htun P, et al. Impact of inflammatory state and metabolic control on responsiveness to dual antiplatelet therapy in type 2 diabetics after PCI: prognostic relevance of residual platelet aggregability in diabetics undergoing coronary interventions. Clin Res Cardiol. 2010;99:743–52. 10.1007/s00392-010-0179-x 20526607

[28] Breet NJ, van Werkum JW, Bouman HJ, Kelder JC, Ruven HJ, Bal ET, et al. Comparison of platelet function tests in predicting clinical outcome in patients undergoing coronary stent implantation. JAMA. 2010;303:754–62. 10.1001/jama.2010.181 20179285

[29] Price MJ, Endemann S, Gollapudi RR, Valencia R, Stinis CT, Levisay JP, et al. Prognostic significance of post-clopidogrel platelet reactivity assessed by a point-of-care assay on thrombotic events after drug-eluting stent implantation. Eur Heart J. 2008;29:992–1000. 10.1093/eurheartj/ehn046 18263931

[30] Stone GW, Witzenbichler B, Weisz G, Rinaldi MJ, Neumann FJ, Metzger DC, et al., ADAPT-DES Investigators. Platelet reactivity and clinical outcomes after coronary artery implantation of drug-eluting stents (ADAPT-DES): a prospective multicentre registry study. Lancet. 2013;382:614–23. 10.1016/S0140-6736(13)61170-8 23890998

